# Optical products for refractive error and low vision

**Published:** 2011-12

**Authors:** Kovin S Naidoo, Yashika I Maharaj, Vivasan Pillay, Sebastian Fellhauer

**Affiliations:** Global Programmes Director, International Centre for Eyecare Education (ICEE); Africa Chair, International Agency for the Prevention of Blindness (IAPB), Director: African Vision Research Institute (AVRI). Email: k.naidoo@icee.org; Global Programmes Officer, ICEE; Postgraduate Student, AVRI; Business Development Manager, ICEE; Social Enterprise Co-ordinator, ICEE

**Figure:**
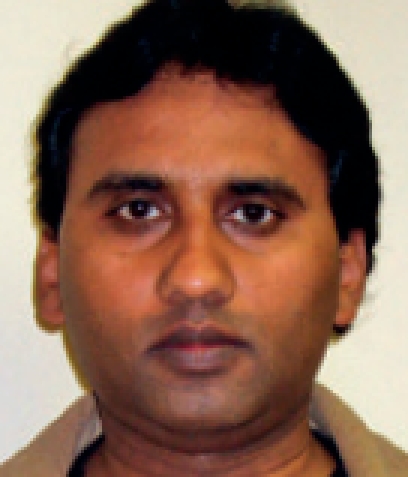
Kovin S Naidoo

**Figure:**
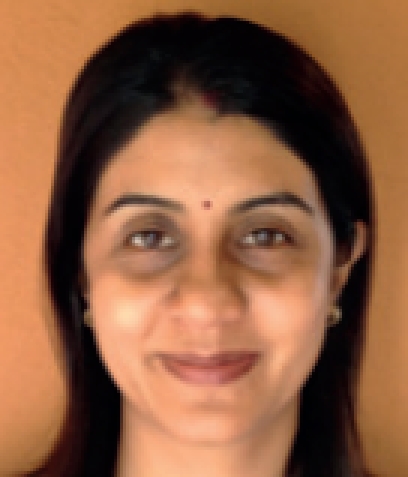
Yashika I Maharaj

**Figure:**
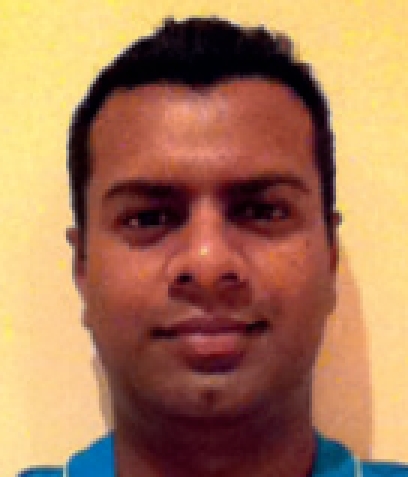
Vivisan Pillay

**Figure:**
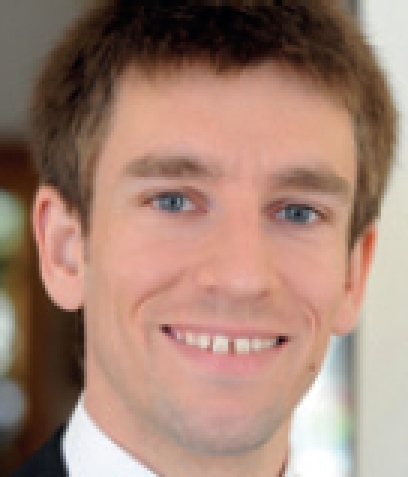
Sebastian Fellhauer

Large numbers of people, mostly in low-and middle-income countries, continue to be blind or visually impaired due to uncor-rected refractive error or cannot function because they lack access to appropriate low vision aids. Numerous challenges have been cited as the reason for the lack of efficient refractive error and low vision services. These include a lack of appropriate human resources, inadequate infrastructure, and lack of optical products such as spectacles and low vision devices.

This article will focus on the optical products required for the efficient delivery of refractive error and low vision services, and provide insight into how they can be managed effectively to ensure a quality service. You can consult the IAPB Standard List (see page 30) for suggestions regarding the optical products you may require at your facility as well as recommended suppliers.

## Finding the right supplier

To have an efficient operation and be effective requires finding the right supplier. Price and location are two key criteria when choosing suppliers.

**Figure:**
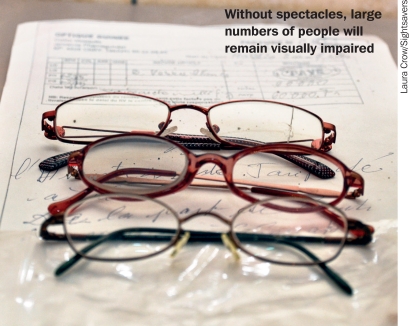


Whilst it may be tempting to use the supplier with the cheapest prices, make sure that products are of an appropriate quality. Obtain recommendations from others who have purchased from the supplier and ask for help from someone who is able to assess the product samples.

Carefully consider the effect the location of your supplier will have on costs. Imported products can be cheaper, but the total cost may be more than that of local products once costs for freight, customs, and duties are included.

**‘Price and availability are two key criteria when choosing suppliers’**

It is also important to get to know more than one supplier. This means that other suppliers can deliver when your preferred supplier is out of stock or unable to supply, for whatever reason.

In the public sector, it is important to find a supplier who understands the philosophy and objectives of your organisation. Social enterprises who conduct operations in the optical industry are usually best positioned to meet such needs as their motive for existing is to make a social impact. Although they conduct commercial transactions, they have a vested interest in assisting community-based and public health organisations to fulfil their objectives.

**Figure 1.**
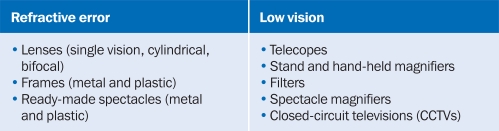
Key optical products for refractive error & low vision

The most widely known social enterprises that supply optical products are the Global Resource Centre and the Hong Kong Society for the Blind (see opposite). Alternatively, if you are part of a non-profit organisation providing refractive error and low vision services, you can invite your optical suppliers to support your work by supplying products at a discount.

## Planning what to stock

### Know your customers

The number of potential customers in your local population should be an indicator of the quantities of stock you will need. Consider the size of the population who will have access to your services. How many people are likely to need spectacles? You can look at data from neighbouring districts, countries, or regions if none are available for your area.

Also consider the different age groups in the population. The needs of an ageing population differ significantly from those of a younger population. Also consider any facial characteristics that are more prevalent in your area so that you can stock the appropriate shapes and sizes of spectacle frames.

### Know what your customers need

Stock levels of optical products should take account of the prevalence of specific eye conditions and the needs of the customers. For example, the sales figures for the Global Resource Centre indicate that ready-made reading spectacles account for approximately 18% of all sales to several public sector hospitals and clinics in Kwazulu-Natal, South Africa. In all likelihood, this is because the ready-made spectacles respond to customer needs on several levels: it is appropriate for a common eye condition in that population (presbyopia), it is immediately available (no waiting times or repeat appointments required), and the price is affordable.

### Know what your customers can afford

The ability of the targeted population to pay for spectacles and low vision devices should determine the particular category of frames, lenses, ready-made spectacles, and low vision devices you stock. If the population consists mainly of people with little money to spend, then it is best to keep a large volume of budget products. If there is a mix, also stock more expensive products for those who have more to spend. In a programme that aims to be financially sustainable by providing products at a cost to the customer, the profits made on middle- to high-end products can be used to subsidise the cost of spectacles or devices for the very poor.

### Know what your customers like

This is probably the most ignored factor as the prevailing thinking is that people with less money are not conscious of their appearance. If you do not take this into consideration, patients may buy a pair of spectacles but decide to not to wear them because they are not fashionable or because they fear being ridiculed.

### Know what sells

To successfully be able to observe and respond to sales trends, you will need systems that track the sale of products. There are many computer software packages that can provide these trends in the form of schedules and graphs based on your daily sales. Even if these are not available to you, the rate at which some frames are depleted and the frequency with which they are ordered will give an indication of what is sold most often. Should the trends show high sales for particular styles, this should be reflected in what you buy: order the most of what you sell the most.

Having focused discussions with small groups of patients about their preferences is another popular method of understanding current and potential sales trends. Try to find out what frame styles, if included, could potentially be popular.

## Ordering optical products

Ordering must be planned well in advance, else you could run out of stock and potentially lose sales. However, ordering and keeping too much stock should also be avoided because money will be tied up in stock that could otherwise have been used in other parts of your operations.

Be aware of public holidays and other periods that your supplier may be unavailable, and factor these into your ordering strategy.

High-powered lenses fall out of the range that is available off-the-shelf and must be made on demand by optical laboratories. These lenses can cost a lot more, particularly in low- and middle-income countries; partly because there are so few of these laboratories.

To keep costs down, identify a manufacturing laboratory in your country or region, negotiate a price based on your projected demand, and choose a cost-effective means of delivery.

## Sources of optical products

### The Global Resource Centre (www.iceegrc.org)

The Global Resource Centre was set up by ICEE to procure refractive error and low vision products for non-governmental organisations and governments. The main aim of this initiative is to reduce the costs of optical products to create greater affordability. The GRC operates an online ordering system and provides the following products:

- Frames (plastic and metal)
- Lenses (spherical, sphero-cylindrical, and bifocal)
- Reading glasses (plastic and metal)
- Refraction equipment
- Low vision aids, in collaboration with the Hong Kong Society for the Blind. To increase access in Africa, the GRC procures these in bulk from the Hong Kong Society for the Blind and is able to supply products at affordable prices.

The GRC is in the process of setting up regional distribution centres to ensure a faster delivery time.

In addition, a partnership is being established with a major logistics company. This will ensure that delivery costs are kept to a minimum and that small orders will be possible.

### Hong Kong Society for the Blind (www.hksb.org.hk)

The Hong Kong Society for the Blind operates the VISION 2020 Low Vision Resource Centre which was set up by IAPB in the 1990s.

The centre's purpose is to centralise the purchase and distribution of quality low vision resources to low- and middle-income countries.

A wide range of visual assessment charts, refraction equipment, training materials, and low vision devices, are available, including:

- Telescopes
- Spectacle magnifiers
- Magnifiers (stand, handheld, bar)
- Closed-circuit televisions (CCTVs)

Further information about the resource centre and its comprehensive product range is available on the website.

**FROM THE FIELD: Supplying spectacles in Papua New Guinea**

**Figure:**
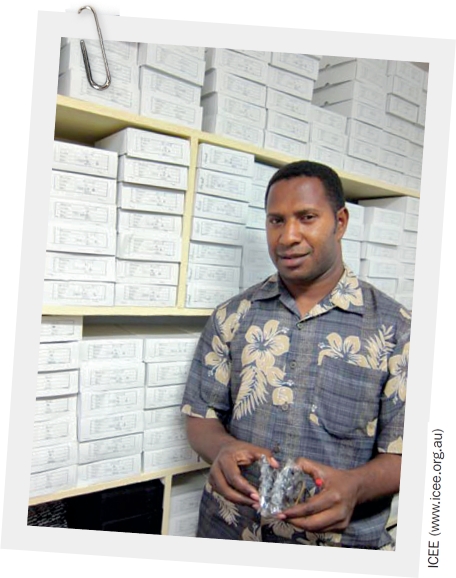

As Papua New Guinea's national spectacles supply system co-ordinator, Moses oversees the spectacle supply chain that serves six eye care vision centres and eight spectacle supply units around the country.

Supplying spectacles to fourteen locations can create logistical challenges. To overcome barriers and ensure that each location has spectacles required to provide patients with the most efficient care, Moses completes monthly reports, talks to everyone involved about what they need and how things are going, and makes certain that ordering and shipping procedures are followed at all times.

“The best thing about my job is that I'm able to meet people at all levels in the hospitals I visit,” said Moses.

**‘I'm able to meet people at all levels in the hospitals I visit’**

The Papua New Guinea eye care vision centres are addressing the lack of eye care services in the country by providing free eye examinations and affordable spectacles. The eye centres are an initiative by International Centre for Eye Care Education (ICEE).

